# Liraglutide and not lifestyle intervention reduces soluble CD163 after comparable weight loss in obese participants with prediabetes or type 2 diabetes mellitus

**DOI:** 10.1186/s12933-024-02237-8

**Published:** 2024-04-29

**Authors:** Helene Grannes, Thor Ueland, Paola Simeone, Rossella Liani, Maria Teresa Guagnano, Pål Aukrust, Annika E. Michelsen, Kåre Birkeland, Augusto di Castelnuovo, Francesco Cipollone, Agostino Consoli, Bente Halvorsen, Ida Gregersen, Francesca Santilli

**Affiliations:** 1 Research Institute for Internal Medicine, Oslo University Hospital Rikshospitalet, Sognsvannsveien 20, 0372 Oslo, Norway; 2 Institute of Clinical Medicine, Faculty of Medicine, University of Oslo, Oslo, Norway; 3 Thrombosis Research and Expertise Centre, University of Tromsø, Tromsø, Norway; 4 Department of Medicine and Aging, and Center for Advanced Studies and Technology (CAST), “G. d’Annunzio” University of Chieti-Pescara, Chieti, Italy; 5 Section of Clinical Immunology and Infectious Diseases, Oslo University Hospital Rikshospitalet, Oslo, Norway; 6 Department of Endocrinology, Morbid Obesity and Preventive Medicine, Oslo University Hospital, Oslo, Norway; 7 Mediterranea Cardiocentro, Naples, Italy

**Keywords:** GLP-1 analogue, Weight loss, Immune cells, T2DM, Obesity

## Abstract

**Background:**

The GLP-1 receptor agonist liraglutide is used to treat hyperglycemia in type 2 diabetes but is also known to induce weight loss, preserve the beta cell and reduce cardiovascular risk. The mechanisms underlying these effects are however still not completely known. Herein we explore the effect of liraglutide on markers of immune cell activity in a population of obese individuals with prediabetes or newly diagnosed type 2 diabetes mellitus.

**Method:**

Plasma levels of the monocyte/macrophage markers, soluble (s)CD163 and sCD14, the neutrophil markers myeloperoxidase (MPO) and neutrophil gelatinase‐associated lipocalin (NGAL),the T-cell markers sCD25 and T-cell immunoglobulin mucin domain-3 (sTIM-3) and the inflammatory marker TNF superfamily (TNFSF) member 14 (LIGHT/TNFSF14) were measured by enzyme-linked immunosorbent assays in obese individuals with prediabetes or diabetes diagnosed within the last 12 months, prior to and after comparable weight loss achieved with lifestyle changes (n = 20) or liraglutide treatment (n = 20), and in healthy subjects (n = 13).

**Results:**

At baseline, plasma levels of the macrophage marker sCD163, and the inflammatory marker LIGHT were higher in cases as compared to controls. Plasma levels of sCD14, NGAL, sTIM-3 and sCD25 did not differ at baseline between patients and controls. After weight reduction following lifestyle intervention or liraglutide treatment, sCD163 decreased significantly in the liraglutide group vs. lifestyle (between-group difference p = 0.023, adjusted for visceral adipose tissue and triglycerides basal values). MPO and LIGHT decreased significantly only in the liraglutide group (between group difference not significant). Plasma levels of MPO and in particular sCD163 correlated with markers of metabolic dysfunction and inflammation. After weight loss, only sCD163 showed a trend for decreased levels during OGTT, both in the whole cohort as in those of liraglutide vs lifestyle group.

**Conclusion:**

Weight loss following treatment with liraglutide was associated with reduced circulating levels of sCD163 when compared to the same extent of weight loss after lifestyle changes. This might contribute to reduced cardiometabolic risk in individuals receiving treatment with liraglutide.

**Supplementary Information:**

The online version contains supplementary material available at 10.1186/s12933-024-02237-8.

## Introduction

Liraglutide is an analogue of glucagon-like peptide-1 (GLP-1) widely used in the treatment of type 2 diabetes (T2DM) and has been shown to reduce body weight [1]. Further, liraglutide is reported to lower cardiovascular and total mortality in patients with T2DM and high cardiovascular risk [2], but the mechanisms are not fully understood. Macrophages, neutrophils and T-cells express the GLP-1 receptor, and thus GLP-1 and its analogues have the potential to affect a wide spectrum of immune cells [3–5]. Liraglutide affects immune cells in obesity mouse models as well as in human and mouse cells in vitro [6–8]. In clinical trials, liraglutide reduces levels of various inflammatory cytokines such as tumor necrosis factor (TNF) and interleukin (IL)-6 in overweight and obese individuals with T2DM [9–11]. We have previously shown that the inflammatory marker *soluble suppression of tumorigenesis-2* (sST2) was decreased after weight loss induced by liraglutide treatment, but not after comparable weight loss due to lifestyle changes [12]. Interestingly, in the same cohort, liraglutide achieved a more pronounced reduction in visceral adipose tissue (VAT) and improvement in beta cell function, independently of weight loss [13]. Monocyte/macrophages are known to be involved in both adipose tissue and islet inflammation, however, the effect of liraglutide treatment on immune cells is still not fully clarified, and it is uncertain whether the anti-inflammatory effects of liraglutide is mainly related to weight reduction.

In the present study, we challenged the idea that immune cells such as monocyte/macrophages, Tcells or neutrophils may be interesting cellular targets of liraglutide, thus contributing to alterations in systemic, adipose tissue and islet cell inflammation. Analyzing plasma from a previous study, comparing liraglutide and lifestyle intervention [13], we explored the regulation of markers reflecting activation of neutrophils: myeloperoxidase (MPO) and neutrophil gelatinase‐associated lipocalin (NGAL), T-cells: soluble (s)CD25 and T-cell immunoglobulin mucin domain-3 (sTIM-3) and monocyte/macrophages: sCD14 and sCD163, before and after similar weight loss induced by liraglutide treatment or lifestyle-changes only.

## Methods

### Subjects and study design

The current study was a post hoc analysis using stored serum and plasma samples from a randomized, controlled, parallel-arm study designed to assess the effects of an equal degree of weight loss, achieved by either lifestyle changes or liraglutide, on cardiometabolic variables in obese subjects with impaired glucose tolerance (IGT) and/or impaired fasting glucose (IFG) or early T2DM. The protocol and patient characteristics have been previously described [13]. In brief, 62 obese patients with prediabetes (IFG and IGT) or early T2DM were enrolled at the Obesity and Diabetes Clinics of Chieti University Hospital. In addition, 13 subjects, without obesity, diabetes mellitus or prediabetes and not on pharmacological treatment, were enrolled as controls. The patients were randomized 1:1 to receive liraglutide or lifestyle counseling. Study medication was supplied to the research pharmacy by Novo Nordisk as liraglutide 6.0 mg/mL in 3-mL prefilled pen injectors. Liraglutide treatment was administered daily by subcutaneous injection at bedtime with an initial dose of 0.6 mg/day (first week) and titrated over a 3-week period to doses of 1.2 mg daily (second week) and 1.8 mg daily (third week) based on the clinical response and side effects. The nonattainment of the 1.8 mg dose level did not constitute a withdrawal criterion. The participants in the liraglutide arm were encouraged to continue with their existing dietary and exercise habits in addition to liraglutide. The second arm consisted of an intensive lifestyle intervention. The lifestyle-arm participants received two initial days of education on the “Mediterranean diet” and food label education. The aim was a diet with an average of 30% lipids, minimum 15% protein, and maximum 10% of simple sugars, with focus on fibre rich foods, nuts, legumes, and fish rich in omega-3 fatty acids. In addition, they were recommended to consume 10 g of dark chocolate daily, as well as to reduce the intake of salt. Regarding physical activity they were recommended three hours of physical activity per week and two of the three hours were scheduled exercise with the intervention team. Both groups had regular check-ins with the intervention team, but the lifestyle group had in addition first weekly, then biweekly, and lastly monthly consultations with the team to discuss how they were doing with the lifestyle program, and to help keep the motivation up until the goal of 7% weight loss was achieved. Participants in both groups continued with their assigned treatment until they lost 7% of their initial body weight (calculated on the basis of body weight at baseline visit at the time of randomization). Six patients did not achieve this amount of weight loss within 15 months after randomization and were excluded, in addition to 16 participants that dropped out, leaving n = 20 in each treatment arm.

All study visits and procedures took place at the Clinical Research Center within Department of Medicine and Aging, Center for Advanced Studies and Technology (CAST), University of Chieti, Italy. Each patient provided written informed consent to participate, and the Protocol was approved by the Ethics Committee of the University of Chieti, and the Regional Ethical Committee in South-Eastern Norway approved the import in Norway of blood for laboratory assessments.

### Blood sampling

Venous blood samples (EDTA platelet-poor plasma) were collected at inclusion in the study and after the achievement of the 7% weight loss goal. At both visits an oral glucose tolerance test (OGTT) was performed and blood samples were taken before (T0), and 60, 90 and 120 min after a 75 g glucose load. The β cell secretion during an OGTT was estimated by applying a minimal model of glucose-induced insulin secretion to the glucose and C-peptide curves of each subject, as previously described in detail [14]. In addition, we evaluated another OGTT-based measure of B-cell function: the insulin secretion-sensitivity index-2 (ISSI-2) (defined as the ratio of the area-under-the-insulin-curve to the area-under-the-glucose curve, multiplied by the Matsuda index) [15]. All samples were frozen at − 80 °C for subsequent biochemical measurements.

### Biochemical measurements

Plasma levels of immune cell markers sCD163, sCD14, MPO, NGAL, sTIM-3 and sCD25 were measured by DuoSet enzyme-linked immunosorbent assays from R&D Systems (Stillwater, MN). The inflammatory marker LIGHT/tumor necrosis factor super family member 14 (TNFSF14) was measured with a Quantikine enzyme-linked immunosorbent assays from R&D Systems (Stillwater, MN). All were analyzed in a 384-format using a combination of a SELMA pipetting robot (Analytik Jena AG, Jena, Germany) and a BioTek dispenser/washer (BioTek Instruments, Winooski, VT). Absorption was read at 450 nm by using an EIA plate reader (BioTek Instruments) with wavelength correction set to 540 nm. Samples from all patients and controls were run on the same 384-well plate. Calculated limit of the detection (3*SD + OD of 0-standard) was 0.45 ng/mL (sCD163), 0.23 ng/mL (sCD14), 0.086 ng/mL (MPO), 0.035 ng/mL (NGAL), 0.0093 ng/mL (sTIM-3), 23 pg/mL (sCD25) and 10.4 pg/mL (LIGHT), respectively. The inter- and intra-assay coefficients of variation were < 10%.

### Statistical analysis

In a study with 20 patients in each treatment arm (liraglutide vs. lifestyle), we were able to detect, by the end of the treatment period, a genuine difference in the mean response between the experimental and control arms, equivalent to one standard deviation of a not pre-specified continuous outcome. This was achieved with a power of 0.9 and a Type I error probability of 0.05. Comparisons of variables between groups (liraglutide versus lifestyle versus controls) and between arms (liraglutide versus lifestyle advice) were performed by χ2 test or Mann–Whitney U test. Spearman rank correlation test was used to assess relationships among continuous variables. All tests were two-tailed. All calculations were carried out using SPSS (SPSS, Chicago, IL, USA).

## Results

### Baseline characteristics

Clinical and biochemical baseline characteristics of the study subjects have been previously presented [12, 13, 16] and relevant variables are shown in Table 1. Patients randomized to liraglutide treatment and lifestyle intervention were similar on most parameters, except for triglycerides (TG), waist circumference and visceral adipose tissue (VAT) being higher in the liraglutide arm. Compared to healthy controls, both patient groups had a lower age, higher BMI, lower CRP higher total- and LDL cholesterol, and lower HDL cholesterol. In the liraglutide group, 10 (50%) subjects had IFG or IGT and in the lifestyle group 13 (65%) subjects had IFG or IGT.

**Table 1:** Clinical and biochemical baseline characteristics of study participants

Variable	Healthy Controls (n = 13)	Liraglutide (n = 20)	Lifestyle (n = 20)	Liraglutide versus Lifestyle p-value	Healthy Subjects versus Liraglutide p-value	Healthy Subjects versus Lifestyle p-value
Age (years)	66.0 (58–69)	55 (48–63)	52 (50–57)	0.481	0.010	0.005
Gender (male), n (%)	7 (53)	11 (55)	10 (50)	1.000	1.000	1.000
BMI (kg/m^2^)	22.8 (21.5–26.6)	36.7 (34.7–40.9)	35.0 (31.3–40.3)	0.244	< 0.001	< 0.001
Weight (Kg)	78.0 (61.5–86.0)	109 (95.0–115.0)	96 (86.0–106.0)	0.056	< 0.001	0.001
T2DM, n (%)	0 (0)	10 (50)	7 (35)	0.523	0.002	0.027
Waist (cm)	NA	116.5 (112.0–128.5)	110.0 (100.4–119.2)	0.040	–	–
WHR	NA	1.0 (0.9–1.0)	0.9 (0.9–1)	0.321	–	–
SAT (cm2)	NA	434.1 (317.9–527.2)	374.9 (254.2–455.3)	0.311	–	–
VAT (cm2)	NA	324.2 (257.0–386.9)	254.5 (180.2–318.9)	0.046	–	–
Systolic BP (mmHg)	NA	144.5 (130–153)	134.0 (122.2–143.2)	0.144	–	–
Diastolic BP (mmHg)	NA	83.0 (78.0–87.5)	80.0 (70.0–83.7)	0.315	–	–
Hypertension, n (%)	NA	17 (85)	12 (60)	0.155	–	–
Dyslipidemia, n (%)	NA	9 (45)	10 (50)	1.000	–	–
CVD, n (%)	NA	1 (5)	5 (25)	0.182	–	–
Carotid stenosis, n (%)	NA	0 (0)	4 (20)	0.106	–	–
Microvascular disease, n (%)	NA	0 (0)	0 (0)	–	–	–
Total cholesterol (mmol/l)	5.2 (4.6–6.3)	4.4 (3.6–5.0)	4.4 (3.8–4.6)	0.337	0.024	0.003
LDL cholesterol (mmol/l)	2.79 (2.56–3.15)	2.45 (1.76–3.26)	2.58 (1.99–3.00)	0.715	0.144	0.187
HDL cholesterol (mmol/l)	1.8 (1.7–2.3)	1.2 (1.0–1.4)	1.1 (1.0–1.4)	0.668	0.001	< 0.001
Triglycerides (mmol/l)	1.00 (0.60–1.38)	1.4 (0.9–2.2)	1.0 (0.8–1.3)	0.026	0.024	0.490
Fasting plasma glucose (mmol/l)	NA	5.2 (4.9–5.9)	5.3 (5.0–5.7)	0.989	–	–
1-h postload plasma glucose (mmol/L)	NA	10.6 (9.2–11.4)	10.6 (8.6–11.2)	0.16	**–**	**–**
2-h postload plasma glucose (mmol/L)	NA	8.7 (7.9–10.7)	8.1 (6.3–10.2)	0.17	**–**	**–**
HbA1c (%)	5.5 (5.3–5.6)	5.95 (5.6–6.7)	6.1 (5.6–6.5)	0.862	< 0.001	< 0.001
HbA1c (mmol/mol)	37 (34–38)	42 (38–50)	43 (38–48)	0.862	< 0.001	< 0.001
Fasting plasma insulin (uU/ml)	NA	13.4 (9.62–20.9)	10.7 (7.5–21.7)	0.394	–	–
1-h postload plasma insulin (uU/ml)	NA	72.7 (31.7–102.8)	78.9 (55.5–140.0)	0.130	–	–
2-h postload plasma insulin (uU/ml)	NA	76.9 (42.9–100.7)	76.3 (55.3–123.3)	0.170	–	
C-reactive protein (mg/dl)	1.15 (0.8–2.7)	0.45 (0.3–0.9)	0.3 (0.1–0.5)	0.354	0.003	0.001
NAFLD (grade)	NA	31.5 (14.0–45.5)	32.0 (17.5–45.7)	0.860	–	–
Beta-index	NA	3.41 (2.58–5.06)	4.34 (2.98–5.29)	0.142	–	–
WBC × 10^3^ /uL	NA	5.90 (5.10–7.30)	5.65 (5.23–6.27)	0.387		
HOMA-IR	NA	3.45 (2.19–4.96)	2.57 (1.87–5.25)	0.482		
Matsuda index	NA	2.84 (2.21–4.19)	2.90 (1.95–4.52)	0.892		
IL-10 (pg/mL)	NA	35.86 (21.75–60.72)	30.62 (10.18–45.48)	0.678		
Leptin (ng/mL)	NA	18.27 (10.85–46.49)	25.11 (13.44–44.52)	0.871		
TNF (pg/mL)	NA	1.10 (0.94–1.44)	1.23 (0.98–1.83)	0.911		
Metformin, n (%)	NA	20(100)	20 (100)	1.000	–	–
ACE-I, n (%)	NA	4 (20)	3 (15)	1.000	–	–
ARBs, n (%)	NA	7 (35)	6 (30)	1.000	–	–
Diuretics, n (%)	NA	7 (35)	5 (25)	0.731	–	–
B-block, n (%)	NA	7 (35)	4 (20)	0.480	–	–
CCA, n (%)	NA	0 (0)	1 (5)	1.000	–	–
Statins, n (%)	NA	2 (10)	5 (25)	0.407	–	–
Fibrates, n (%)	NA	0 (0)	0 (0)	–	–	–
Omega 3, n (%)	NA	1 (5)	0 (0)	1.000	–	–
Proton Pump Inhibitors, n (%)	NA	3 (15)	3 (15)	1.000	–	–
ASA, n (%)	NA	1 (5)	3 (15)	0.605	–	–

Data are median (25th–75th percentile)

BMI: Body mass index, T2DM: Type 2 diabetes mellitus, WHR: Waist-hip ratio, SAT: Subcutaneous-adipose-tissue, VAT: Visceral-adipose-tissue, BP: blood pressure,, CVD: Cardiovascular disease, NAFLD: Non-alcoholic fatty liver disease, WBC: White blood count, HOMA-IR: homeostatic model assessment insulin resistance, TNF: Tumour necrosis factor, ACE-I: ACE-inhibitors, ARBs: Angiotensin receptor blockers, B-block: Beta-blockers, CCA: Calcium channel antagonists, ASA: Acetylsalicylic acid

Data analyzed with Mann Whitney test

### Baseline comparisons of soluble immune cell markers between participants with obesity and healthy controls

At baseline, plasma levels of the macrophage marker sCD163, but not sCD14, was higher in patients as compared to healthy subjects (Table 2). The neutrophil marker MPO was higher in the lifestyle group, compared to the liraglutide group, but when compared to controls they were not statistically different (Table 2). There were no significant differences in plasma levels of the neutrophil marker NGAL, or of the T-cell activation markers, sTIM-3 and sCD25, when comparing cases and controls at baseline (Table 2).

**Table 2:** Baseline concentrations of soluble immune cell activity markers

Variable	Healthy Controls (n = 13)	Liraglutide (n = 20)	Lifestyle (n = 20)	Liraglutide versus Lifestyle p-value	Healthy Subjects versus Liraglutide p-value	Healthy Subjects versus Lifestyle p-value
sCD163 (ng/ml)	268.0 (591.0–115.0)	393.8 (210.0–757.0)	449.1 (149.0–449.1)	0.923	0.001	0.013
sCD14 (ng/ml)	1036.8 (479.0–1331.0)	1057.0 (264.0–1683.0)	1093.3 (808.0–1631.0)	0.920	0.231	0.944
MPO (ng/ml)	14.1 (2.8–24.1)	14.4 (2.6–27.2)	18.9 (10.8–33.5)	0.030	0.081	0.840
NGAL (ng/ml)	83.9 (115.0–64.5)	79.1 (56.8–79.1)	91.6 (59.8–160.5)	0.102	0.492	0.519
sTIM-3 (ng/ml)	3.0 (0.84–4.2)	3.8 (2.7–3.8)	3.5 (1.8–3.5)	0.808	0.316	0.165
sCD25 (pg/ml)	482.2 (298.0–942.9)	513.8 (267.2–513.8)	501.8 (285.7–1068.3)	0.883	0.330	0.888

Data are median (25th–75th percentile)

MPO: Myeloperoxidase, NGAL: Neutrophil gelatinase‐associated lipocalin, sTIM-3: T-cell immunoglobulin mucin domain-3.

Data analyzed with Mann Whitney test

### Baseline sCD163 and MPO correlate with markers of metabolic dysfunction and inflammation

In the study group as a whole, baseline levels of sCD163 correlated positively with several metabolic parameters such as BMI (rho = 0.432, p = 0.006), C-peptide (rho = 0.410, p = 0.009), insulin (rho = 0.340, p = 0.034), total cholesterol (rho = 0.358, p = 0.025), leptin (rho = 0.481, p = 0.002) and HOMA-IR (rho = 0.389, p = 0.014), and negatively with Matsuda index (rho = − 0.337, p = 0.042). Baseline levels of sCD163 also correlated positively with other markers related to inflammation, i.e., C-reactive protein (CRP, rho = 0.337, p = 0.042), IL-10 (rho = 0.559, p = 0.002), and total leukocyte counts (rho = 0.493, p = 0.001) as well as with non-alcoholic fatty liver disease (NAFLD) prior to intervention (rho = 0.356, p = 0.026, Additional file 1: Table S1).

Baseline MPO showed a negative correlation with waist-to-hip ratio (WHR, rho = − 0.345, p = 0.031) and beta-index (rho = − 0.356, p = 0.024, Additional file 1: Table S2).

### Liraglutide treatment improved metabolic parameters compared to lifestyle intervention

All study participants except one attained the 1.8 mg dose level through-out the study period. The amount of weight loss was prespecified by the protocol to 7% of initial body weight and did not differ between the groups. Median time to predefined weight loss was 4.8 months and did not differ between the two treatment arms [13]. Concomitant therapy was unchanged during the follow-up. At the end of the intervention period (i.e. after achievement of the weight loss target) both groups experienced a reduction in several metabolic parameters, including BMI, HbA1c, fasting plasma insulin, disposition index, as well as CRP as a reliable marker of systemic inflammation, with the decrease in VAT and beta-index being more pronounced in the liraglutide arm (Table 3) [13]. The liraglutide arm showed decreased systolic blood pressure and total cholesterol, and improved beta cell function, as assessed by beta-index, and glucose tolerance, indicated by reduced fasting glucose and 1- and 2-h post load glucose levels (Table 3). On the other hand, the lifestyle intervention group showed a significant decrease in 2-h post load insulin levels, which was not significant in the liraglutide group (Table 3). There were, however, no associations between changes in the inflammatory markers (sCD163 and MPO) and changes in any of metabolic parameters (Additional file 1: Table S4).

**Table 3:** Clinical and laboratory characteristics of obese patients before and after liraglutide or lifestyle-induced weight loss intervention

Variable	Pre-Liraglutide	Post-Liraglutide	p-value	Pre-Lifestyle	Post-Lifestyle	p-value
BMI (kg/m^2^)	36.73 (34.69–40.89)	33.93 (31.40–37.94)	< 0.001	35.0 (31.3–40.3)	32.5 (29.0–37.1)	< 0.001
Waist (cm)	116.5 (112.0–128.5)	110.0 (104.25–120.75)	< 0.001	110.0 (100.4–119.2)	106.0 (97.2–112.7)	0.001
WHR	0.97 (0.92–1.04)	0.98 (0.92–1.00)	0.586	0.9 (0.9–1)	0.9 (0.9–1)	0.778
Systolic BP (mmHg)	144.5 (130–153)	133.0 (122.0–144.5)	0.029	134.0 (122.2–143.2)	133.0 (125.0–143.0)	0.679
Diastolic BP (mmHg)	83.0 (78.0–87.5)	78.5 (69.25–83)	0.079	80.0 (70.0–83.7)	80.0 (77.0–86.0)	0.477
Total cholesterol (mmol/L)	4.4 (3.6–5.02)	3.9 (3.4–4.6)	< 0.001	4.4 (3.8–4.6)	4.2 (3.7–4.6)	0.507
HDL cholesterol (mmol/L)	1.2 (1.0–1.4)	1.15 (1.0–1.4)	0.085	1.1 (1.0–1.4)	1.2 (0.9–1.3)	0.397
Triglycerides (mmol/L)	1.4 (0.9–2.2)	1.5 (0.9–1.7)	0.286	1.0 (0.8–1.3)	90.5 (1.0–1.5)	0.314
Amylase (U/L)	56.5 (53.5–70.75)	67.5 (46.7–82.2)	0.647	62.5 (52.5–77.2)	74.5 (55.2–90.7)	0.046
Lipase (U/L)	105.0 (66.2–117.5)	132.0 (99.0–223.0)	0.004	134.5 (66.5–173.2)	118.5 (79.0–172.0)	0.881
Fasting plasma glucose (mmol/L)	5.2 (4.9–5.9)	4.9 (4.5–5.2)	0.001	5.3 (5.0–5.7)	4.9 (4.6–5.2)	0.057
1- hour- post load prandial glucose (mmol/L)	10.6 (9.2–11.4)	9.0 (7.1–10.0)	< 0.001	10.0 (8.6–11.2)	8.7 (7.8–9.9)	0.097
2-h –post load prandial glucose (mmol/L)	8.7 (7.9–10.7)	7.2 (5.1–9.9)	0.001	8.1 (6.3–10.1)	7.7 (5.7–10.4)	0.390
HbA1c (%)	5.95 (5.62–6.70)	5.65 (5.40–5.97)	< 0.001	6.1 (5.6–6.5)	5.6 (5.4–6.1)	0.001
HbA1c (mmol/mol)	41 (38–50)	38 (36–42)	< 0.001	43 (38–48)	38 (36–43)	0.001
Fasting plasma insulin (uU/ml)	13.35 (9.62–20.92)	9.75 (6.67–15.12)	0.015	10.7 (7.5–21.7)	8.9 (6.3–11.1)	0.001
2-h-post load prandial insulin (uU/ml)	80.4 (47.3–107.3)	56.7 (33.4–106.1)	0.654	86.5 (55.9–147.6)	55.6 (33.1–118.2)	0.014
Creatinine (mg/dL)	0.70(0.63–0.81)	0.7 (0.6–0.9)	0.802	0.8 (0.7–0.9)	0.8 (0.7–0.9)	0.360
Total bilirubin (mg/dl)	0.60(0.42–0.90)	0.56(0.50–0.76)	0.879	0.6 (0.4–0.8)	0.6 (0.4–0.8)	0.813
C-reactive protein (mg/dL)	0.45 (0.27–0.86)	0.29 (0.09–0.56)	0.004	0.30 (0.1–0.5)	0.25 (0.2–0.3)	0.018
AST (U/L)	29.0 (24.2–39)	22.0 (19.2–25.5)	< 0.001	33.0 (27.5–43.5)	24.0 (20.0–33.7)	< 0.001
ALT (U/L)	41.0 (36.2–46.5)	31.0 (26.2–37.0)	< 0.001	50.0 (33.2–66.5)	35.0 (28.2–49.0)	0.001
SAT (cm^2^)	434.1 (317.9–527.2)	421.0 (258.2–481.8)	0.001	374.9 (254.2–455.3)	294.3 (210.6–403.8)	< 0.001
VAT (cm^2^)	324.2 (257.0–386.9)	274.6 (183.9–321.2)	< 0.001	254.5 (180.2–318.9)	231.6 (171.3–290.7)	0.017
Beta index (pmol.min^−2^.m^−2^ BSA)	3.41 (2.58–5.06)	4.77 (3.38–5.5)	0.001	4.34 (2.98–5.29)	4.79(3.49–5.24)	0.296
Disposition Index	2.1 (1.3–3.2)	3.7 (0.9–6.0)	0.020	2.8 (1.2–6.06)	3.3 (2.3–4.8)	0.227
ISSI-2	118.3 (81.2–158.2)	218.0 (110.0–274.7)	< 0.001	166.7 (106.0–219.1)	187.6 (145.3–218.7)	0.073

Data are median (25th–75th percentile)

BMI: Body mass index, WHR: Waist-hip ratio, BP: Blood pressure, AST: Aspartate transaminase, ALT: Alanine transaminase, SAT: Subcutaneous adipose tissue, VAT: Visceral adipose tissue, ISSI-2: Insulin secretion-sensitivity index-2

Data analyzed with Mann Whitney test

### Liraglutide, but not lifestyle changes reduce levels of sCD163 and MPO

**Fig. 1:**
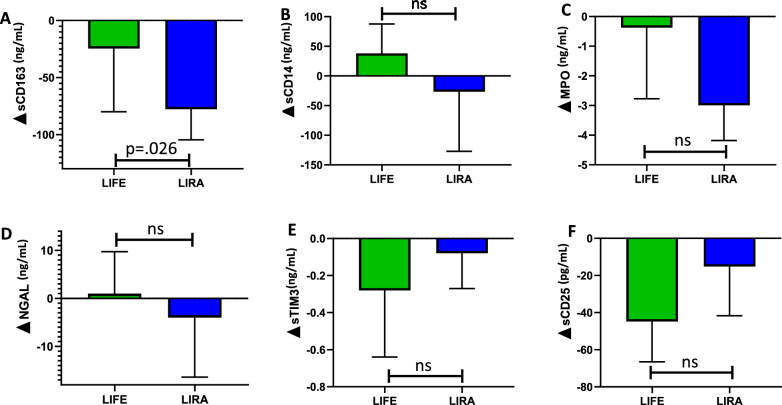
Changes in plasma concentration of soluble immune markers during a liraglutide-(green bar) or lifestyle-induced (blue bar) weight loss intervention. **A** sCD163, (**B**) sCD14, (**C**) MPO, (**D**) NGAL, (**E**) sTIM-3 and (**F**) sCD25. LIFE: lifestyle intervention group, LIRA: Liraglutide intervention group, ns: not significant, NGAL: Neutrophil gelatinase‐associated lipocalin, MPO: Myeloperoxidase, sTIM-3: T-cell immunoglobulin mucin domain-3, ∆: Change from baseline (pre) to post-intervention

At the end of the intervention period, we observed a significant reduction in sCD163 levels in the liraglutide arm (∆ = 87, SD = 115, p = 0.001), but not in the lifestyle arm (∆ = 24, SD = 85), with a significant between-group difference in ∆sCD163 also when adjusted for basal VAT and basal triglycerides values (p = 0.026) (Fig. 1A). In contrast, levels of sCD14 as an additional marker of monocyte/macrophage activation, were not affected by intervention in any of the two arms (Fig. 1B). Weight loss induced a significant decrease in MPO levels in the group receiving liraglutide (p = 0.048) (Fig. 1C), and not in the lifestyle intervention group, but the difference in decreases between arms was not statistically significant. For the other neutrophil markers (i.e., NGAL) and the T-cell markers (i.e., sTIM-3 and sCD25) no significant between arm difference was observed during the intervention. (Fig. 1D–F).

### sCD163 levels were regulated during an oral glucose tolerance test, after weight loss intervention

**Fig. 2:**
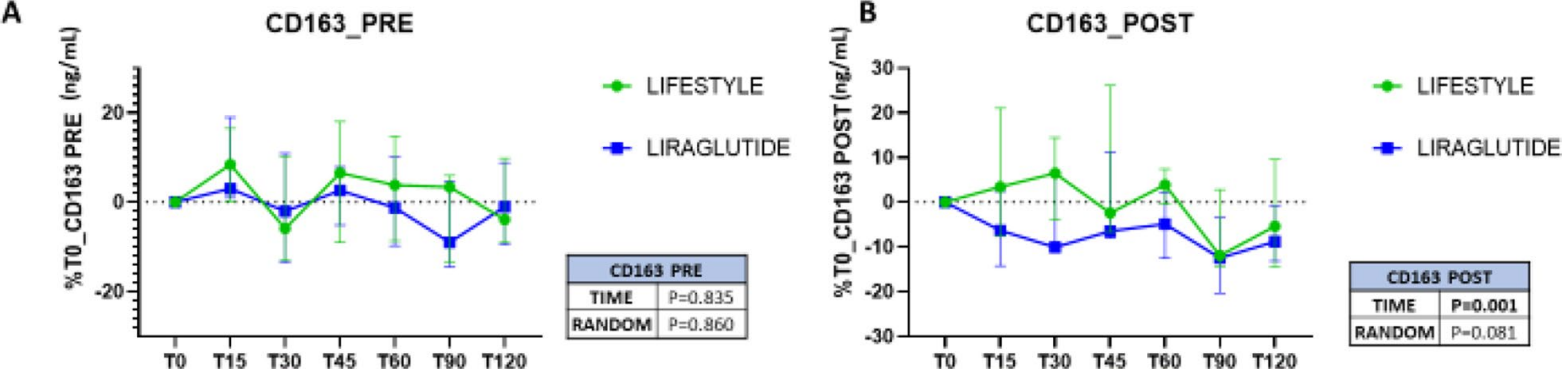
Percentage change from baseline concentrations of sCD163 during an oral glucose tolerance test, comparing lifestyle and liraglutide intervention groups. (**A**) pre, and (**B**) post intervention

A 75 g oral glucose load given before the intervention, did not show any change in levels of sCD163 over a period of 120 min (p = 0.835) and there were, as expected, no differences in response between the treatment groups at baseline (p = 0.860) (Fig. 2A). After intervention, however, we observed a reduction in the levels of sCD163 in the whole study population, after the glucose load over time (p = 0.001). This effect was more pronounced in patients randomized to liraglutide compared to the lifestyle arm, although the difference between arms was not statistically significant (p = 0.081, Fig. 2B). Interestingly, and in concordance with the baseline correlations between plasma glucose and sCD163, during the OGTT post intervention, the sCD163 AUC after glucose-load was significantly and directly related to the glucose AUC only in the lifestyle arm (data not shown), suggesting that liraglutide directly affects sCD163, independently of blood glucose rise. No change in any of the other variables studied was observed during OGTT neither before nor after weight loss.

### Liraglutide, but not lifestyle changes reduce levels of LIGHT

**Fig. 3:**
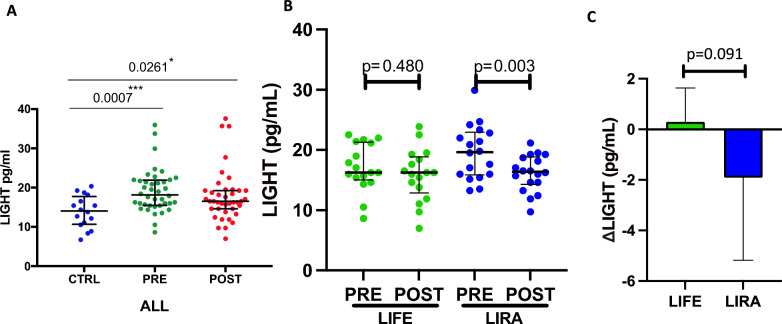
Plasma concentrations of LIGHT in participants before and after liraglutide or lifestyle-induced weight loss intervention. (**A)** Comparing concentrations pre- and post-intervention of all participants, (**B**) Comparing pre- and post-intervention concentrations in liraglutide and lifestyle treatment groups, (**C**) Comparing change in concentrations from baseline between treatment groups. LIGHT: TNF superfamily (TNFSF) member 14, LIFE: Lifestyle treatment group, LIRA: Liraglutide treatment group, ∆: Change from baseline (pre) to post-intervention

We have previously shown that the inflammatory cytokine LIGHT/TNFSF14 is increased in patients with T2DM and can induce beta cell death and impair insulin secretion [17]. Monocyte/macrophages are important cellular sources of LIGHT and based on the regulation of sCD163 in the liraglutide arm, we therefore analyzed LIGHT levels in the study group. At baseline, LIGHT was significantly increased in the patient cohort, as compared to controls (Fig. 3A), and indeed, LIGHT showed a positive correlation with sCD163 (rho = 0.417, p = 0.001) in the study group as a whole. Further, LIGHT correlated with total leukocyte counts (rho = 0.395, p = 0.017) and with ISSI-2 at baseline (rho = − 0.321, p = 0.044) (Additional file 1: Table S3). After intervention, LIGHT was reduced in the liraglutide group (p = 0.003), but not in the lifestyle group, the difference between treatment arms where however not statistically significant (Fig. 3B, C).

## Discussion

Liraglutide is an acylated glucagon-like peptide-1 analogue with 97% amino acid homology with endogenous GLP-1 and greatly protracted action. It is widely used for the treatment of T2DM and administered by subcutaneous injection once daily [18]. GLP-1 analogues were initially developed to treat T2DM patients, in whom the effects upon glycemia and, also weight loss, were evident. Recently, the latter effect of these drugs has received much attention [19].

CD163 is a member of the scavenger receptor superfamily, categorized into class B, and its soluble form, sCD163, is regarded as a marker of activated M2 macrophages, and thus potentially a systemic marker reflecting a counteracting mechanism of pro-inflammatory activation of tissue-resident macrophages [20]. sCD163 is cleaved upon activation by a myriad of stimuli, and plasma levels are most likely due to a combination of CD163 expression, altered clearance rate and increased shedding [21]. Increased sCD163 has been shown in obesity [22, 23], metabolic dysregulation and visceral adiposity [24], and is considered a marker of insulin resistance and future T2DM [25, 26]. In keeping with this, we found that baseline sCD163 was associated with several markers of metabolic dysfunction (e.g., a negative association with insulin sensitivity, as measured by Matsuda index) and with markers of inflammation such as CRP. In a recent study, and in agreement with our data, serum levels of sCD163 were higher in patients with obesity and metabolic syndrome as compared to controls [27]. Furthermore, a decrease in serum concentrations of sCD163 and fewer inflammatory macrophages has been previously demonstrated in patients with T2DM treated for 6 months with liraglutide [10], but the present study is, as far as we know, the first to compare levels of immune cell markers after liraglutide treatment and lifestyle intervention with comparable weight loss.

The mechanisms for the sCD163-reducing effect of liraglutide are not known, but we have previously shown improved beta-cell function and reduced VAT after liraglutide treatment, and reduced macrophagic sCD163 might be connected to both these events [13]. Indeed, macrophages are the primary immune cells involved in obesity-associated islet inflammation in both mice and humans [28, 29]. Along these lines, in the same cohort of obese subjects with prediabetes or early diabetes we showed that individuals treated with liraglutide experienced a larger reduction in VAT [10], as compared to individuals who achieved the same extent of weight loss receiving lifestyle intervention. As VAT is regarded as more inflammatory than the subcutaneous adipose tissue (SAT) compartment [30], macrophages from VAT may be a primary source of sCD163 and VAT reduction with liraglutide treatment may mirror reduced macrophage inflammation, as assessed by decreased circulating sCD163. In contrast, Pastel et al*.* showed that treatment with liraglutide, compared to dietary restriction-based weight loss, increased adipose tissue inflammation, measured as CD14, MCP-1, TNF and IL-6 gene expression and concluded that despite a stronger improvement of glycemic control, liraglutide was not effective in amelioration of obesity-associated adipose tissue dysfunction [31]. However, in that study, the degree of weight reduction between the groups was not equal and gene expression may not necessarily reflect the protein levels, which both could be significant confounding factors. Nonetheless, the reduction in sCD163 in the liraglutide arm and not in the lifestyle intervention arm with comparable weight loss, as well as lack of association between changes in sCD163 and changes in any of metabolic parameters such as BMI, HbA1c, fasting plasma insulin and Matsuda index, support an immune-modulating effect of this GLP-1 analog at least partly independent of the weight loss and related metabolic changes.

Obesity promotes local replication of islet-resident macrophages and recruits circulating monocytes [29]. Islet macrophages in obese mice have been shown to dampen beta cell insulin secretion and promote beta cell proliferation [28]. Thus, targeting islet macrophages is a potential therapeutic approach to modulate beta cell function and prevent development of T2DM. In this regard we previously reported, in patients with T2DM, increased circulating levels of the inflammatory mediator LIGHT/TNFSF14 largely derived from activated monocytes and platelets [17]. We further showed that receptors for LIGHT on islet cells are upregulated and can induce beta cell death and impair insulin secretion from human pancreatic islets in vitro [17], thus contributing to occurrence of overt diabetes and its progression. Interestingly, in the present cohort LIGHT and sCD163 were both increased in obese subjects vs. controls, directly related with each other at baseline, and both were significantly reduced after liraglutide treatment but not during life-style intervention. Thus, we can speculate that GLP-1RAs may act on circulating monocyte/islet macrophages, decrease the release of LIGHT, thus reducing the extent of systemic and local inflammation, leading to improved beta cell function, as assessed by beta-index [13].

MPO has also been shown to be causally linked to development of obesity and insulin resistance [32]. In humans, MPO is upregulated in obesity, independently of T2DM status [33]. In contrast to these findings, baseline MPO was herein not significantly increased in individuals with obesity, as compared to controls, and was negatively correlated with WHR. Nevertheless, the liraglutide group experienced a decrease in MPO levels during weight reduction which was not seen in the lifestyle intervention group. Thus, our data may suggest specific anti-inflammatory effects of liraglutide in T2DM, independent of weight reduction, potentially also involving attenuated neutrophil responses. In cardiovascular safety trials in T2DM patients, with most individuals presenting with cardiovascular disease and excess weight, GLP-1RAs decreased cardiovascular risk [34]. In the case of the LEADER study, major adverse cardiovascular events (MACE) decreased by 13 percent with liraglutide [2]. The mechanism behind the cardiovascular protection observed with human GLP-1RAs in T2DM is not fully known. Carotid plaque inflammation is mitigated in patients treated with GLP-1RAs [35], pointing to reduced inflammation as a possible mechanism underlying the reduction in cardiovascular risk. Gaining insight into the complex mechanisms through which GLP-1RAs produce their anti-inflammatory effects will improve our understanding of their therapeutic potential and facilitate the creation of new anti-inflammatory approaches [36]. Both MPO and LIGHT are associated with increased cardiovascular disease risk and in particular sCD163 is associated with both incidence and death from cardiovascular disease [37–41], and thus reduced levels of these could be one of the mechanisms contributing to the cardioprotective effects of liraglutide.

The present study has some limitations such as a relatively low number of participants. Despite the small sample size, we adjusted for VAT and triglycerides, values that were significantly different between arms at baseline, but we intentionally refrained from conducting further adjustments which may not yield reliable results and could potentially introduce spurious associations. Nonetheless, the lack of correction for several potentially confounding factors is a limitation of the present study. Univariate correlation analyses cannot be used to make assumptions about a causal relationship, and analyzing a large number of variables increases the likelihood of false positive correlations. Another limitation is that there were no measures of diet nor exercise compliance during the intervention.

Finally, our understanding of the inherent functions of the soluble immune cell markers is lacking. Strengths of our study includes the randomized design of the study and the comparable weight reduction between the intervention groups, excluding the confounding effect of different weight loss on the markers in study, as well as the detailed characterization of the metabolic features of participants.

Nevertheless, these data provide novel insight to the regulation of immune cell markers after liraglutide treatment, and a potential mechanism for the observed metabolic benefits seen with liraglutide as compared with lifestyle intervention for the treatment of T2DM and obesity. These data are also interesting in relation to the more widely use of GLP-1 analogs in obese patients to obtain weight reduction independent on the presence of diabetes. Moreover, reduced levels of sCD163, LIGHT and MPO could reflect the observed decrease in VAT, preservation of beta cell function as well as cardiovascular protection by liraglutide treatment, but this needs to be confirmed in larger studies and validation cohorts.

### Supplementary Information

**Additional file 1: Table S1. **Spearman correlations between sCD163 and clinical and biochemical parameters at baseline in the total study population that participated in a liraglutide or lifestyle-induced weight loss intervention. **Table S2. **Spearman correlations between MPO and clinical and biochemical parameters at baseline in the total study population that participated in a liraglutide or lifestyle-induced weight loss intervention. **Table S3. **Spearman correlations between LIGHT and clinical and biochemical parameters at baseline in the total study population that participated in a liraglutide or lifestyle-induced weight loss intervention. **Table S4. **Spearman correlations between changes in sCD163, MPO and LIGHT with changes in selected metabolic parameters in the total study population that participated in a liraglutide or lifestyle-induced weight loss intervention.

## Data Availability

The datasets analysed during the study are available from the project leader Francesca Santilli, MD, PhD, upon reasonable request (email: f.santilli@unich.it).

## Abbreviations

GLP-1: Glucagon-like peptide-1
MPO: Myeloperoxidase
LIGHT: TNF superfamily (TNFSF) member 14
OGTT: Oral glucose tolerance test
T2DM: Type 2 diabetes mellitus
TNF: Tumor necrosis factor
IGT: Impaired glucose tolerance
IFT: Impaired fasting glucose
EDTA: Ethylenediaminetetraacetic acid
TG: Triglycerides
VAT: Visceral adipose tissue
SAT: Subcutaneous adipose tissue
BMI: Body mass index
LDL: Low-density lipoprotein
HDL: High-density lipoprotein
HOMA-IR: Homeostatic model assessment insulin resistance
CRP: C-reactive protein
NAFLD: Non-alcoholic fatty liver disease
WHR: Waist-Hip Ratio
CVD: Cardiovascular disease
MCP-1: Monocyte chemoattractant protein-1
IL-6: Interleukin 6
