# Supplementary material for: Liraglutide and not lifestyle intervention reduces soluble CD163 after comparable weight loss in obese participants with prediabetes or type 2 diabetes mellitus
Source: Cardiovasc Diabetol. 2024 Apr 29;23:146. doi: 10.1186/s12933-024-02237-8 (PMC11059692; doi:10.1186/s12933-024-02237-8)
Supplement: Supplementary file 1 — Additional file 1: Table S1. Spearman correlations between sCD163 and clinical and biochemical parameters at baseline in the total study population that participated in a liraglutide or lifestyle-induced weight loss intervention. Table S2. Spearman correlations between MPO and clinical and biochemical parameters at baseline in the total study population that participated in a liraglutide or lifestyle-induced weight loss intervention. Table S3. Spearman correlations between LIGHT and clinical and biochemical parameters at baseline in the total study population that participated in a liraglutide or lifestyle-induced weight loss intervention. Table S4. Spearman correlations between changes in sCD163, MPO and LIGHT with changes in selected metabolic parameters in the total study population that participated in a liraglutide or lifestyle-induced weight loss intervention. [file 12933_2024_2237_MOESM1_ESM.docx]

**Additional data**

**Additional file 1: Table S1:** Spearman correlations between sCD163 and clinical and biochemical parameters at baseline in the total study population that participated in a liraglutide or lifestyle-induced weight loss intervention

| **Spearman Correlation** | **rho** | **95% confidence interval** | **P (two-tailed)** |
| --- | --- | --- | --- |
| **sCD163 vs. BMI** | **.432** | **.125 to .663** | **.006** |
| **sCD163 vs. C-peptide** | **.410** | **.0993 to .648** | **.009** |
| **sCD163 vs. insulin** | **.340** | **.0178 to .598** | **.034** |
| **sCD163 vs. total cholesterol** | **.358** | **.0384 to .611** | **.025** |
| **sCD163 vs. leptin** | **.482** | **.187 to .697** | **.002** |
| **sCD163 vs. HOMA-IR** | **.389** | **.0737 to .633** | **.014** |
| **sCD163 vs. Matsuda index** | **-.406** | **-.645 to -.0945** | **.010** |
| **sCD163 vs. CRP** | **.337** | **.00415 to .602** | **.042** |
| **sCD163 vs. IL-10** | **.480** | **.111 to .733** | **.011** |
| **sCD163 vs. MCP-1/CCL2** | **.464** | **.133 to .708** | **.007** |
| **sCD163 vs. WBC** | **.493** | **.200 to .704** | **.001** |
| **sCD163 vs. NAFLD** | **.356** | **.0365 to .610** | **.026** |
| sCD163 vs. WHR | -.110 | -.410 to .213 | .503 |
| sCD163 vs. Beta-index | -.149 | -.442 to .176 | .363 |
| sCD163 vs. TNF | .237 | -.091 to .515 | .150 |
| **sCD163 vs. LIGHT** | **.417** | **.167 to .612** | **.001** |
| sCD163 vs. creatinine | -.176 | -.464 to .149 | .283 |
| sCD163 vs. MPO | .079 | -.193 to .339 | .569 |
| sCD163 vs. ISSI-2 | -.158 | -.450 to .166 | .333 |

BMI = body mass index, HOMA-IR = homeostatic model assessment insulin resistance, CRP = C-reactive protein, MCP-1 = monocyte chemoattractant protein-1, CCL2 = chemokine (C-C motif) ligand 2, WBC = white blood count, NAFLD = non-alcoholic fatty liver disease, WHR = waist-hip ratio, TNF = tumor necrosis factor, LIGHT = TNF superfamily (TNFSF) member 14, MPO = myeloperoxidase, ISSI-2 = insulin secretion-sensitivity index-2

**Additional file 1: Table S2:** Spearman correlations between MPO and clinical and biochemical parameters at baseline in the total study population that participated in a liraglutide or lifestyle-induced weight loss intervention

| **Spearman Correlation** | **rho** | **95% confidence interval** | **P (two-tailed)** |
| --- | --- | --- | --- |
| MPO vs. BMI | .126 | -.198 to .423 | .443 |
| MPO vs. C peptide | -.213 | -.493 to .112 | .192 |
| MPO vs. insulin | -.090 | -.393 to .232 | .584 |
| **MPO vs. total cholesterol** | **.385** | **.075 to .622** | **.015** |
| MPO vs. leptin | .213 | -.112 to .493 | .191 |
| MPO vs. HOMA-IR | -.104 | -.405 to .219 | .524 |
| MPO vs. Matsuda Index | .115 | -.208 to .414 | .482 |
| MPO vs. CRP | .159 | -.175 to .457 | .346 |
| MPO vs. IL-10 | .155 | -.219 to .486 | .413 |
| MPO vs. MCP-1/CCL2 | .119 | -.240 to .448 | .513 |
| MPO vs. WBC | .151 | -.174 to .444 | .356 |
| MPO vs. NAFLD | -.073 | -.379 to .248 | .657 |
| **MPO vs. WHR** | **-.345** | -.593 to -.029 | **.031** |
| **MPO vs. beta-index** | **-.356** | -.574 to -.000 | **.024** |
| **MPO vs. TNF-α** | **.364** | -.011 to .572 | **.023** |
| **MPO vs. LIGHT** | **.349** | -.013 to .485 | **.027** |
| **MPO vs. creatinine** | **-.373** | -.060 to -.018 | **.019** |
| MPO vs. ISSI-2 | -.252 | -.523 to .072 | .121 |

BMI = body mass index, HOMA-IR = homeostatic model assessment insulin resistance, CRP = C-reactive protein, MCP-1/ CCL2 = monocyte chemoattractant protein-1/chemokine (C-C motif) ligand 2, WBC = white blood count, NAFLD = non-alcoholic fatty liver disease, WHR = waist-hip ratio, TNF = tumor necrosis factor, LIGHT = TNF superfamily (TNFSF) member 14, MPO = myeloperoxidase, ISSI-2 = insulin secretion-sensitivity index-2

**Additional file 1: Table S3:** Spearman correlations between LIGHT and clinical and biochemical parameters at baseline in the total study population that participated in a liraglutide or lifestyle-induced weight loss intervention

| **Spearman Correlations** | **rho** | **95% confidence interval** | **P (two-tailed)** |
| --- | --- | --- | --- |
| LIGHT vs. BMI | .0259 | -.314 to .360 | .881 |
| LIGHT vs. C-peptide | .105 | -.242 to .427 | .544 |
| sCD163 vs. insulin | .233 | -.114 to .529 | .172 |
| LIGHT vs.total cholesterol | .0261 | -.314 to .360 | .880 |
| LIGHT vs. leptin | .195 | -.152 to .500 | .254 |
| LIGHT vs. HOMA-IR | .224 | -.123 to .522 | .190 |
| LIGHT vs. Matsuda index | -.0478 | -.379 to .294 | .782 |
| LIGHT vs. CRP | .00246 | -.340 to .344 | .989 |
| LIGHT vs. IL-10 | .137 | -.284 to .514 | .513 |
| LIGHT vs. MCP-1/CCL2 | .0101 | -.368 to .385 | .958 |
| **LIGHT vs. WBC** | **.395** | **.0665 to .646** | **.017** |
| LIGHT vs. NAFLD | -.264 | -.552 to .0812 | .120 |
| LIGHT vs. WHR | -.190 | -.472 to .130 | .238 |
| LIGHT vs. beta-index | -.201 | -.481 to -.119 | .211 |
| LIGHT vs. TNF | .185 | -.140 to .471 | .258 |
| LIGHT vs. creatinine | -.042 | -.349 to .270 | .793 |
| **LIGHT v. sCD163** | **.417** | **.167 to .612** | **.001** |
| **LIGHT vs. ISSI-2** | **-.321** | **-.256 to -0.003** | **.044** |

BMI = body mass index, CRP = C-reactive protein, MCP-1 = monocyte chemoattractant protein-1, CCL2 = chemokine (C-C motif) ligand 2, WBC = white blood count, NAFLD = non-alcoholic fatty liver disease, WHR = waist-hip ratio, TNF = tumor necrosis factor, LIGHT = TNF superfamily (TNFSF) member 14, MPO = myeloperoxidase, ISSI-2 = insulin secretion-sensitivity index-2

**Additional file 1: Table S4:** Spearman correlations between changes in sCD163, MPO and LIGHT with changes in selected metabolic parameters in the total study population that participated in a liraglutide or lifestyle-induced weight loss intervention.

| Δ sCD163 (ng/ml) vs. | rho | 95% confidence interval | P (two-tailed) |
| --- | --- | --- | --- |
| Δ weight | 0.087 | -0.244 to 0.400 | 0.598 |
| Δ BMI | 0.0409 | -0.287 to 0.360 | 0.805 |
| Δ Systolic BP | 0.12 | -0.217 to 0.431 | 0.474 |
| Δ Diastolic BP | 0.162 | -0.176 to 0.466 | 0.332 |
| Δ HbA1c | 0.165 | -0.169 to 0.464 | 0.317 |
| Δ HOMA-IR | 0.309 | -0.0167 to 0.576 | 0.055 |
| Δ matsuda index | -0.0597 | -0.377 to 0.270 | 0.718 |
| Δ insulin | 0.238 | -0.0932 to 0.522 | 0.144 |
| Δ MPO (ng/ml) vs. | **rho** | **95% confidence interval** | **P (two-tailed)** |
| Δ weight | 0.127 | -0.201 to 0.430 | 0.434 |
| Δ BMI | 0.0809 | -0.246 to 0.391 | 0.62 |
| Δ Systolic BP | -0.143 | -0.447 to 0.190 | 0.384 |
| Δ Diastolic BP | -0.0747 | -0.389 to 0.256 | 0.651 |
| Δ HbA1c | 0.0974 | -0.230 to 0.405 | 0.55 |
| Δ HOMA-IR | -0.0598 | -0.373 to 0.265 | 0.714 |
| Δ matsuda index | 0.00901 | -0.312 to 0.328 | 0.956 |
| Δ insulin | -0.171 | -0.466 to 0.158 | 0.291 |
| Δ LIGHT (pg/ml) vs. | **rho** | **95% confidence interval** | **P (two-tailed)** |
| Δ weight | 0.0314 | -0.314 to 0.370 | 0.858 |
| Δ BMI | -0.000421 | -0.343 to 0.342 | 0.998 |
| Δ Systolic BP | -0.02 | -0.365 to 0.330 | 0.911 |
| Δ Diastolic BP | 0.149 | -0.209 to 0.472 | 0.399 |
| Δ HbA1c | 0.0254 | -0.320 to 0.365 | 0.885 |
| Δ HOMA-IR | -0.235 | -0.535 to 0.116 | 0.173 |
| Δ Matsuda index | 0.305 | -0.0415 to 0.586 | 0.075 |
| Δ insulin | -0.272 | -0.562 to 0.0780 | 0.115 |

∆ = change from baseline (pre) to post-intervention, BMI = body mass index, BP = blood pressure, HOMA-IR = homeostatic model assessment insulin resistance, LIGHT = TNF superfamily (TNFSF) member 14, MPO = myeloperoxidase
